# Effects of different intra canal medicaments on the push out bond strength of endodontic sealers

**DOI:** 10.4317/jced.53522

**Published:** 2017-03-01

**Authors:** Sahar Shakouie, Shahriar Shahi, Mohammad Samiei, Amin-Salem Milani, Mohammad-Frough Reyhani, Sara Paksefat, Mahsa Eskandarinekhad, Negin Ghasemi

**Affiliations:** 1 Associate Professor, Department of Endodontics, Dental Faculty, Tabriz University (Medical Sciences), Tabriz, Iran; 2 Professor, Dental and Periodontal Research Center, Department of Endodontics, Dental Faculty, Tabriz University (Medical Sciences), Tabriz, Iran; 3 Associate Professor, Department of Endodontics, Dental Faculty, Tabriz University (Medical Sciences), Tabriz, Iran; 4 Assistant Professor, Dental and Periodontal Research Center, Department of Endodontics, Dental Faculty, Tabriz University (Medical Sciences), Tabriz, Iran; 5 Associate Professor, Dental and Periodontal Research Center, Department of Endodontics, Dental Faculty, Tabriz University (Medical Sciences), Tabriz, Iran; 6 Assistant Professor, Department of Endodontics, Dental Faculty, Ardabil University (Medical Sciences), Ardabil, Iran; 7 Assistant Professor, Department of Endodontics, Dental Faculty, Tabriz University (Medical Sciences), Tabriz, Iran; 8 Assistant Professor, Dental and Periodontal Research Center, Department of Endodontics, Dental Faculty, Tabriz University (Medical Sciences), Tabriz, Iran

## Abstract

**Background:**

One of the essential properties of the root canal sealers is the adhesion to root canal dentin and their higher bond strength decreases the microleakage. The aim of the present study was to compare the effect of Different Intracanal medicaments on the push out bond strength of AH26 and MTA Fillapex sealers.

**Material and Methods:**

A total of 104 one-rooted extracted human teeth were divided into 4 (n=26) experimental groups. After the cleaning and shaping, the root canals were filled with Ca(OH)2, triantibiotic paste (TAP), Metapex or 2% chlorhexidine gel for two weeks. Then, intracanal medicaments were rinsed away and the samples in the sub-groups were obturated with gutta-percha and AH26 or MTA Fillapex sealers. After two weeks incubation, 2-mm-thick middle section of each root was then subjected to push-out testing. Data were analyzed with two-way ANOVA and LSD test.

**Results:**

With all the intracanal medicaments, the overall mean of bond strength values were significantly higher with AH26 compared to MTA Fillapex (*p*<0.05). With the use of MTA Fillapex the maximum and minimum means of bond strength values were recorded with CHX and Metapex and for AH26 were recorded with Ca(OH)2 and chlorhexidine, respectively.

**Conclusions:**

The bond strengths of sealers to dentin are under the influence of pre-treatment with intracanal medicaments. Under the limitations of the present study, the effect of TAP on the bond strength of endodontic sealers was not negative.

** Key words:**AH26, medicament, MTA Fillapex, push-out bond.

## Introduction

The outcome of endodontic treatment depends on the chemomechanical debridement and disinfection of the root canal as important factors. Bacteria remaining in the root canal system and their proliferation might exert negative effects on the outcome of endodontic treatment (1). Although root canal preparation processes have made a lot of progress in recent years, no single technique can completely debride the root canal system. Therefore, researchers have recommended the use of an intracanal dressing to decrease bacterial counts in the root canals (2). Calcium hydroxide (Ca(OH)2) is widely used as an intracanal dressing due to its antibacterial activity and Chlorhexidine (CHX) is used due to its proper antimicrobial activity and low toxicity (3).

Triantibiotic paste (TAP) is a newly introduced material which is used to disinfect necrotic root canals in immature teeth. It is a combination of three antibiotics: metronidazole, ciprofloxacin and minocycline (4). The published literatures showed that it is a proper disinfectant when used as an intracanal medicament. In addition, *in vivo* and *in vitro* studies have shown that this combination of three antibiotics is effective in eliminating endodontic pathogens (5).

Complete obturation of the root canal system and provision of a proper seal at the root canal walls after preparation of the canals are important aims of endodontic treatments and since it is not possible to achieve a proper seal without using sealers, it is necessary to use a sealer with a high capability to bond to dentin and gutta-percha to achieve a proper seal (6). Based on criteria explained by Grossman, adhesion of a sealer to dentin is one of the most favorable properties because in the first place it eliminates spaces for the passage of fluids between the obturation material and the root canal wall and secondly it prevents displacement of root canal obturation material during subsequent restoration procedures (7,8).

The effect of irrigation solutions and intracanal medicaments on the bond strength of sealers to dentin has been evaluated in a large number of studies. Various studies have shown that use of calcium hydroxide increases the bond strength of endodontic sealers. Previous studies on the effects of TAP and Ca(OH)2 on root dentin showed that these medications result in the demineralization of root canal dentin and alter surface collagen (9,10).

To date, no studies have evaluated the effects of other intracanal medicaments, such as triantibiotic paste and other compositions of Ca(OH)2, such as Metapex, and 2% CHX on the bond strength of MTA Fillapex and AH26 to the root canal wall dentin. This study aimed to evaluate this.

## Material and Methods

The design of this study was approved in Tabriz Dental and Periodontal Research Center’s investigation committee.

A total of 104 extracted single-rooted human teeth were selected and stored in 0.5% chloramine solution. The roots were evaluated in relation to the presence of cracks, caries, restorations, absorption and open apices and were excluded from the study if any of the conditions above was present. Then a diamond disk (Buehler, Lake Bluff, NY) was used under copious water spray to remove tooth crowns to achieve a root length of 15 mm in all the samples. A #15 K-file (Densply, Maillefer) was used to exactly determine the working length. The file was placed in the root canal and when the file tip was just visible at the apical foramen under a magnifying glass (Hong Kong Lumagny, No. 7540) it was measured and 1 mm short of this measurement was registered as the WL.

The root canals were prepared using the step-back technique up to #40 K-Flexofile (Densply, Maillefer). Gates-Glidden drills (Mani, Tochigi, Japan) #4 to #1 were used in the coronal and middle thirds of the root canals for standardization of the root canal space and elimination of its taper to standardize the amount of force applied on the obturation materials. A total of 2 mL of 5.2% NaOCl was used between files with a 27-guage needle in a syringe for root canal irrigation. Finally, the root canals were irrigated with 5 mL of 17% EDTA (Meta Dental, Elmhurst, NY) for 3 minute and 5 mL of distilled water were used as the final rinse. The root canals were dried with paper points.

Subsequently, the teeth were divided into 4 groups (n=26) as follows.

Group 1: A pure mix of Ca(OH)2 (Golchay, Tehran, Iran) was prepared with distilled water at a wt% of 44% and placed in the root canal with the use for a lentulo spiral.

Group 2: Metapex (Meta Dental, Elmhurst, NY) was injected into the root canals with the use of special tips provided by the manufacturer.

Group 3: Triantibiotic paste was prepared by mixing 250 mg of each of the three antibiotics (metronidazole, ciprofloxacin and minocycline) with distilled water and injected into the root canals with a 20-guage needle .

Group 4: A 2% gel of CHX 2% (Endogel, Itapetininga, SP, Brazil) was injected into the root canals using the special syringe pro-vided by the manufacturer.

The coronal access cavity was sealed with Cavisol (Golchai, Tehran, Iran) and the sample were incubated at 37°C and 100% relative humidity for 2 weeks. After 2 weeks, the intracanal medicaments were removed by irrigation with 5 mL of distilled water and use of a #40 K-file and a final rinse with 5 mL of distilled water.

Each group was subdivided into two sub-groups as follows.

Sub-group A: The root canals were obturated with gutta-percha points and AH26 sealer (Dentsply, Konstanz, Germany) using lateral compaction technique.

Sub-group B: The root canals were obturated with gutta-percha points and MTA Fillapex sealer(Angelus, Londrina, PR, Brazil) using the lateral compaction technique.

Then all the samples were sealed with Cavit and incubated at 37°C under 100% relative humidity for 2 weeks, after which the root samples were cut into 2-mm-thick cross-sections in the middle third of the roots perpendicular to the root surface using a disk (Buehler, Lake Bulff, NY). Twenty slices were prepared in each test group. A push-out force was applied with a cylindrical piston measuring 0.8 mm in diameter at a crosshead speed of 1 mm/min, perpendicular to the sample surface using a universal testing machine (Hounsfield Test Equipment, Model HSK-S, Surrey, UK). The maximum force at material displacement was recorded in Newton and converted to MPa using the formula below.

The surface area under load= dentin thickness in the slice × the canal circumference 

Two-way ANOVA was used to evaluate the significance of the effect of medicaments on the push out bond strength. Post hoc LSD test was used to exactly determine the differences between the medicaments. Statistical significance was defined at *P*<0.05.

## Results

 Table 1 presents the mean bond strength values of gutta-percha and sealers in all the study groups. The results showed that with all the intracanal medicaments, the overall mean bond strength of AH26 sealer was significantly higher than that of MTA Fillapex sealer (*p*<0.05) ( Table 1).  Table 2 shows the pairwise comparison of the bond strength of study groups.

**Table 1:**
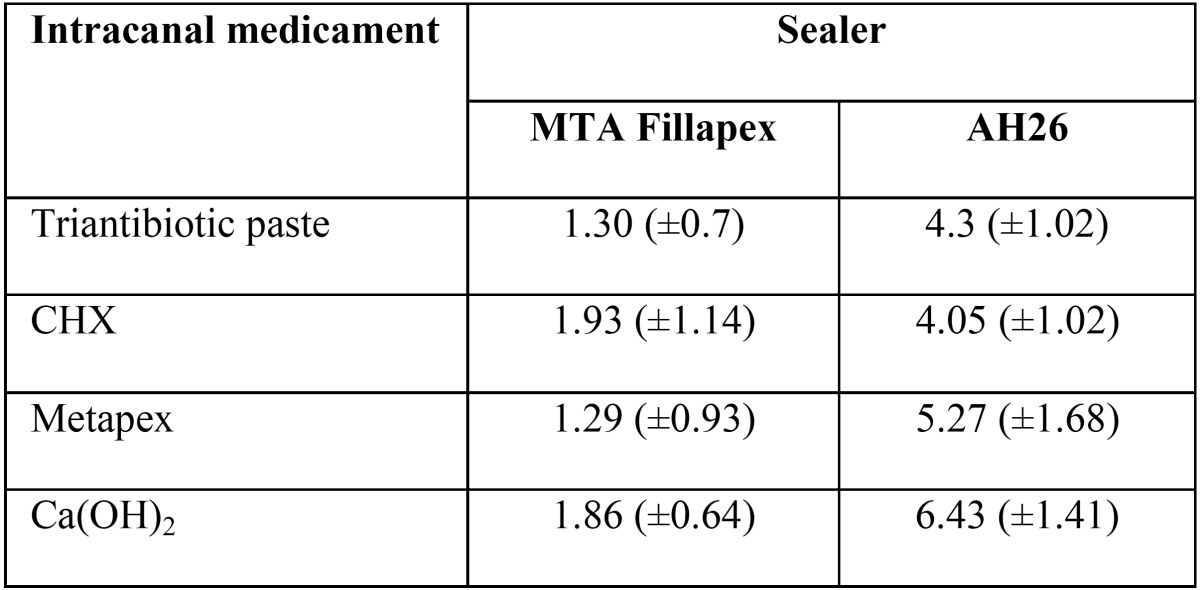
Mean ± SD of bond strength values of the study groups.

**Table 2:**
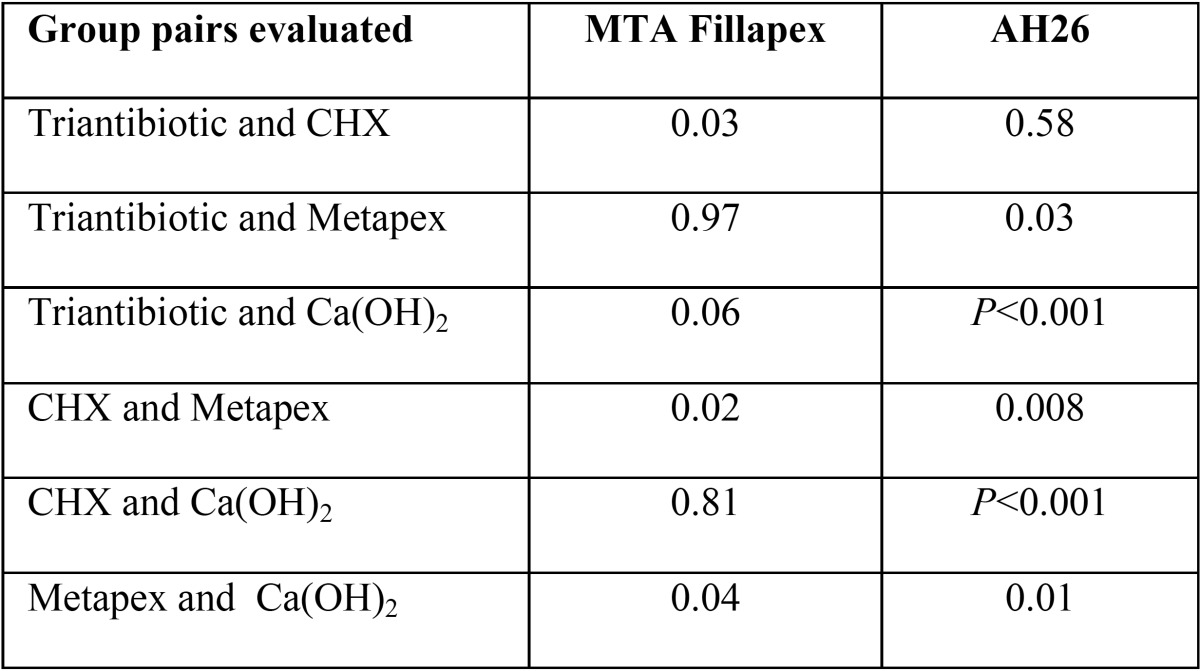
The results of LSD test and *P*-values for the evaluation of differences between the intracanal medicaments with AH26 and MTA Fillapex sealers.

## Discussion

The present study compared the push out bond strength of two endodontic sealers in presence of different intra canal medicaments. Based on the results, AH 26 had higher bond strength than MTA Fillapex. AH 26 and MTA Fillapex had the higher bond strength in presence of Ca(OH)2 and CHX, respectively.

Use of intracanal medicaments between treatment sessions has been recommended to promote intracanal disinfection during endodontic procedures (11,12). Several studies have shown that chemical irrigants and intracanal medicaments can affect the properties of root dentin at the dentin/obturation material interface and the effect of intracanal medicaments on the bond strength between the sealer and dentin appears to be important due to the interaction of epoxy resin-based sealers with root dentin during the polymerization process (7,13,14).

The adhesion of endodontic sealers to root canal dentin is usually evaluated by push-out test. Push-out test is easy to reproduce and to interpret and also for being able to realistically record, at even low levels, the bond strength to dentin. It is also possible to eliminate operator-dependence state of the test by carrying it out by one operator, leading to its popularity (15-17).

There are large numbers of studies underway on intracanal dressings in order to find the best material. Some of the commonly used materials are Ca(OH)2, 2% CHX and TAP.

Calcium hydroxide is widely and routinely used as the most common intracanal medicament. It has been shown that short- and long-term use of Ca(OH)2 in the root canal might exert negative effects on the chemomechanical properties of the root dentin, which might be attributed to its high pH value, with detrimental effects on the surface collagen of root dentin (3). However, since it has been shown in some studies that pure Ca(OH)2 has no effect on resistant microorganisms within the root canal, it is not known as a universal intracanal medication, and attempts are underway to find a more effective medications to eliminate micro-organisms from the root canal system.

A new composition of Ca(OH)2 has recently been introduced, with a trade name of Metapex, which contains Ca(OH)2 plus iodoform and a silicon oil. In a study by Cwikla *et al.*, Metapex exhibited a more potent antibacterial activity within the root canal compared to pure Ca(OH)2 (9). Metapex was selected in the present study because the presence of iodoform and silicone oil in its chemical structure might exert an effect different from that of pure Ca(OH)2 on the bond strength of sealers to dentin.

CHX has been introduced as an irrigation solution and an intracanal medicament and it has been shown that 2% gel of CHX can be effective against microorganisms that are routinely isolated from contaminated canals, resistant to Ca(OH)2 (18).

Another material which was used in the resent study was TAP which contains three antibiotics: metronidazole, ciprofloxacin and minocycline. It is used to disinfect necrotic root canals in immature teeth. *In vitro* and *in vivo* studies have shown that this paste is effective against endodontic pathogens and in the majority of studies it has shown strong effects on bacterial biofilms compared to Ca(OH)2 and CHX (19).

The present study was carried out on intracanal dressings and showed that the mean bond strength with AH26 sealer was higher that of MTA Fillapex; the majority of previous studies have shown that the bond strength of epoxy resin-based sealers, such as AH Plus and AH26, to dentin is higher than that of MTA Fillapex, a sealer with calcium silicate base, which might be attributed to the ability of the epoxide open ring to create a covalent bond with the amino groups of dentin collagen (7,13).

More exact analysis and comparisons of the medicaments showed a higher bond strength with Ca(OH)2 in the AH26 group than the mean bond strength with the use of all the three other materials, consistent with the results of a study by Barbizam *et al.*, who showed the positive effect of Ca(OH)2 on the bond strength of a resin-based sealer, i.e. Epiphany, to dentin. In the Metapex group, both sealers exhibited lower bond strength compared to Ca(OH)2 (2), which might be attributed to the difficulty of removing it completely from the root canal, compared to that of pure Ca(OH)2.

In the CHX group, AH26 sealer exhibited lower bond strength compared to calcium hydroxide. in the MTA Fillapex group, CHX exhibited higher bond strength compared to other materials, consistent with the results of a study by Padro *et al.*, who showed that use of CHX as a final rinse had no negative effect on the bond strength of resin-based sealers because CHX does not produce any smear layer and has no effect on the collagen on root dentin surface (20).

Finally, triantibiotic paste did not exert any effect different from that of CHX in the AH26 sealer groups; however, it exhibited lower bond strength compared to other materials and in the MTA Fillapex group, only in compare with CHX, it exhibited a significant difference.

## Conclusions

Bond strength of MTA Fillapex and AH26 sealers after pretreatment of the root canal with different intracanal medicaments undergoes some alterations and Metapex in general leads to unfavorable outcomes compared to pure Ca(OH)2. The present study is the first study available on the effect of TAP on the bond strength of root canal obturation materials to root dentin and none of the sealer groups in which this paste was used exhibited the least bond strength values.
